# Prolongation of prothrombin time-international normalized ratio by concomitant use of warfarin and combination therapy with lenvatinib and pembrolizumab in a patient with renal cell carcinoma on dialysis: a case report

**DOI:** 10.1186/s40780-026-00598-8

**Published:** 2026-06-13

**Authors:** Shota Torii, Tomoya Narita, Aya Torii-Goto, Takamasa Homma, Hideki Esaki, Takashi Sakakibara, Atsuya Kondo, Norio Takimoto

**Affiliations:** 1 Department of Pharmacy, Kariya Toyota General Hospital, 5-15 Sumiyoshi-cho , Kariya-City, Aichi 448- 8505 Japan; 2 Department of Urology, Kariya Toyota General Hospital, Aichi, 448- 8505 Japan; 3 Department of Pharmacy, College of Pharmacy, Kinjo Gakuin University, Nagoya, 463-8521 Japan

**Keywords:** Lenvatinib and pembrolizumab, Warfarin, Renal cell carcinoma, Dialysis, Drug interaction

## Abstract

**Background:**

Acquired polycystic kidney disease is associated with an increased risk of developing renal cell carcinoma (RCC), which is commonly observed in patients on dialysis and may present with inferior vena cava tumor thrombus. However, there are no prior reports on the use of warfarin for inferior vena cava tumor thrombus in patients on dialysis with RCC receiving combination therapy with lenvatinib and pembrolizumab.

**Case presentation:**

We describe a 64-year-old Japanese man with RCC complicated by tumor thrombus extending into the renal vein and inferior vena cava who was undergoing hemodialysis. Warfarin was initiated at 2 mg/day due to the risk of thrombus progression. His baseline prothrombin time-international normalized ratio (PT-INR) was 1.80 before the initiation of combination therapy with lenvatinib and pembrolizumab. After 8 days of warfarin administration, combination therapy with lenvatinib (10 mg/day) and pembrolizumab (200 mg every 3 weeks) was initiated. Nine days later, the PT-INR elevated to 3.02 despite no change in warfarin dose. The patient remained asymptomatic. Adjusting warfarin to 2–2.5 mg/day and close PT-INR monitoring successfully restored values to the therapeutic range of 1.6–2.6 by day 80, without reducing the lenvatinib dose.

**Conclusions:**

Neither warfarin nor lenvatinib is eliminated by dialysis; therefore, potential pharmacokinetic interactions should be managed with the same caution as in non-dialysis patients. Concomitant use of warfarin and combination therapy with lenvatinib and pembrolizumab in patients with RCC undergoing hemodialysis may enhance anticoagulant effects via multiple interacting factors, including cytochrome P-450-related interactions, systemic inflammation, and hypoalbuminemia. Therefore, careful follow-up, including shorter intervals between outpatient visits (e.g., weekly monitoring), is essential for safe PT-INR management.

## Background

Acquired polycystic kidney disease (APKD) occurs in patients with end-stage renal disease, with an incidence exceeding 50% in patients undergoing dialysis for ≥ 3 years [1, 2]. The risk of developing renal cell carcinoma (RCC) in patients with APKD is approximately 50 times higher than that in the general population [2]. Additionally, inferior vena cava tumor thrombus is observed in approximately 4%–10% of cases of RCC [3, 4], which is considered a risk factor for thrombus formation. Anticoagulant therapy is indicated for prophylaxis when an inferior vena cava filter is inappropriate [5, 6]. The Japanese Guidelines for Pulmonary Thromboembolism, Deep Venous Thrombosis, and Pulmonary Hypertension, the European Society of Cardiology [7], and the CHEST Guidelines [8] recommend the use of direct oral anticoagulants (DOACs) for anticoagulation therapy in pulmonary embolism and deep vein thrombosis. DOACs are favored because they do not require dose adjustment based on blood tests and are associated with fewer bleeding complications. However, DOACs are unsuitable for patients on dialysis. Most DOACs undergo extensive renal excretion, which increases the risk of drug accumulation and bleeding in patients with end-stage renal disease [9]. In contrast, warfarin is considered a viable anticoagulant for patients on dialysis because its systemic clearance is essentially independent of renal function, relying almost entirely on hepatic metabolism via cytochrome P-450 (CYP) 2C9 [10–12]. Notably, warfarin exhibits high plasma protein binding (98%–99%) and primarily binds to albumin [13, 14].

Recently, combination therapy with lenvatinib and pembrolizumab has been used as the first-line treatment for advanced RCC [15]. Lenvatinib is an oral inhibitor of multiple receptor tyrosine kinases, including vascular endothelial growth factor receptors 1–3, fibroblast growth factor receptors 1–4, platelet-derived growth factor receptor α, rearranged during transfection, and KIT [16, 17]. Lenvatinib has been reported to inhibit CYP2C8 and CYP2C9 [18]. Lenvatinib also has a high protein-binding rate (98%–99%) [19, 20] and mainly binds to albumin [21].

In this report, we present a case of a patient with RCC on dialysis who received warfarin as anticoagulant therapy concomitantly with combination therapy with lenvatinib and pembrolizumab.

## Case presentation

**Fig. 1:**
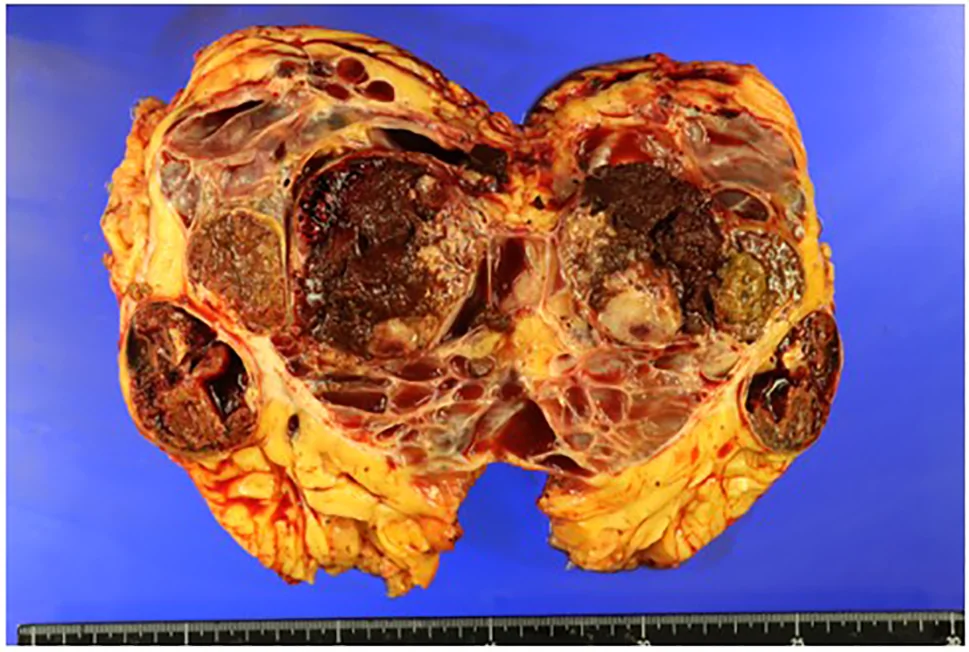
Histological finding of renal cell carcinoma associated with acquired cystic kidney disease. Kidney on dialysis showing multicystic changes and an unclear normal architecture. Part of the kidney was filled with brown muddy material (maximum diameter: 67 mm)

**Fig. 2:**
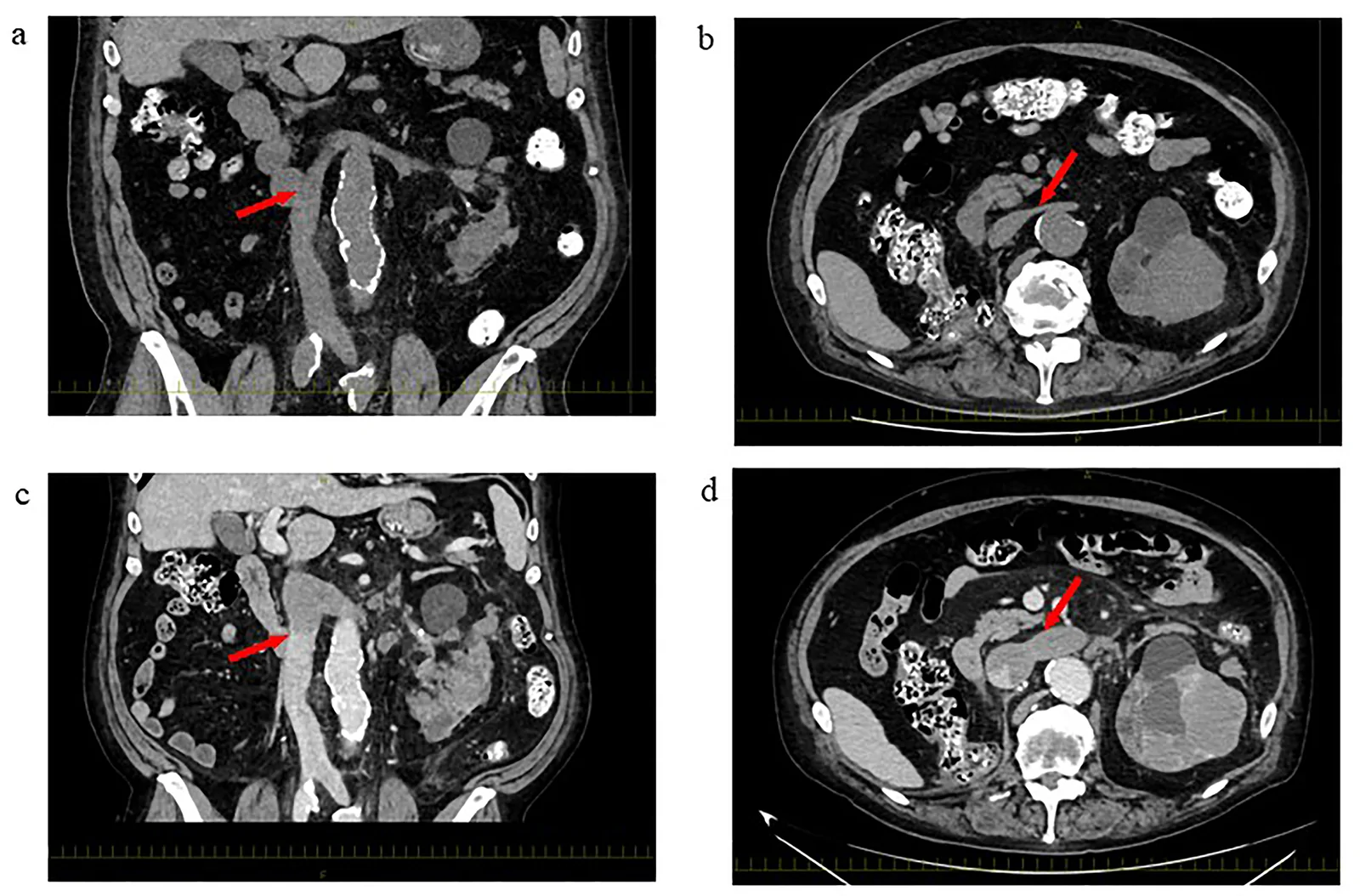
Abdominal CT after radical nephrectomy. (**a**, **c**) Inferior vena cava and (**b**, **d**) left renal vein. (**a**, **b**) No abnormal finding about relapse 4 months after radical nephrectomy. (**c**, **d**) Tumor embolisms in the left renal vein 7 months after radical nephrectomy. CT: computed tomography

A 64-year-old Japanese male patient with a history of spondylosis deformans, bile duct stones, and gastric cancer (post-endoscopic submucosal dissection) presented to our hospital. The patient had been undergoing hemodialysis three times weekly for the past 20 years due to chronic glomerulonephritis. He was diagnosed with right RCC. No tumor was detected in the left kidney, although multiple cysts were observed. The patient underwent radical nephrectomy of the right kidney. Pathological evaluation revealed RCC associated with acquired cystic kidney disease (pathological T1b, Fuhrman grade 3) (Fig. 1). Computed tomography (CT) scan performed 4 months after radical nephrectomy revealed no recurrence (Fig. 2a, b). However, magnetic resonance imaging and contrast-enhanced CT performed 7 months after surgery revealed left RCC with renal vein and inferior vena cava tumor thrombus (Fig. 2c, d). A cardiology consultation was performed due to the possibility of thrombus formation due to the inferior vena cava tumor thrombus.

**Fig. 3:**
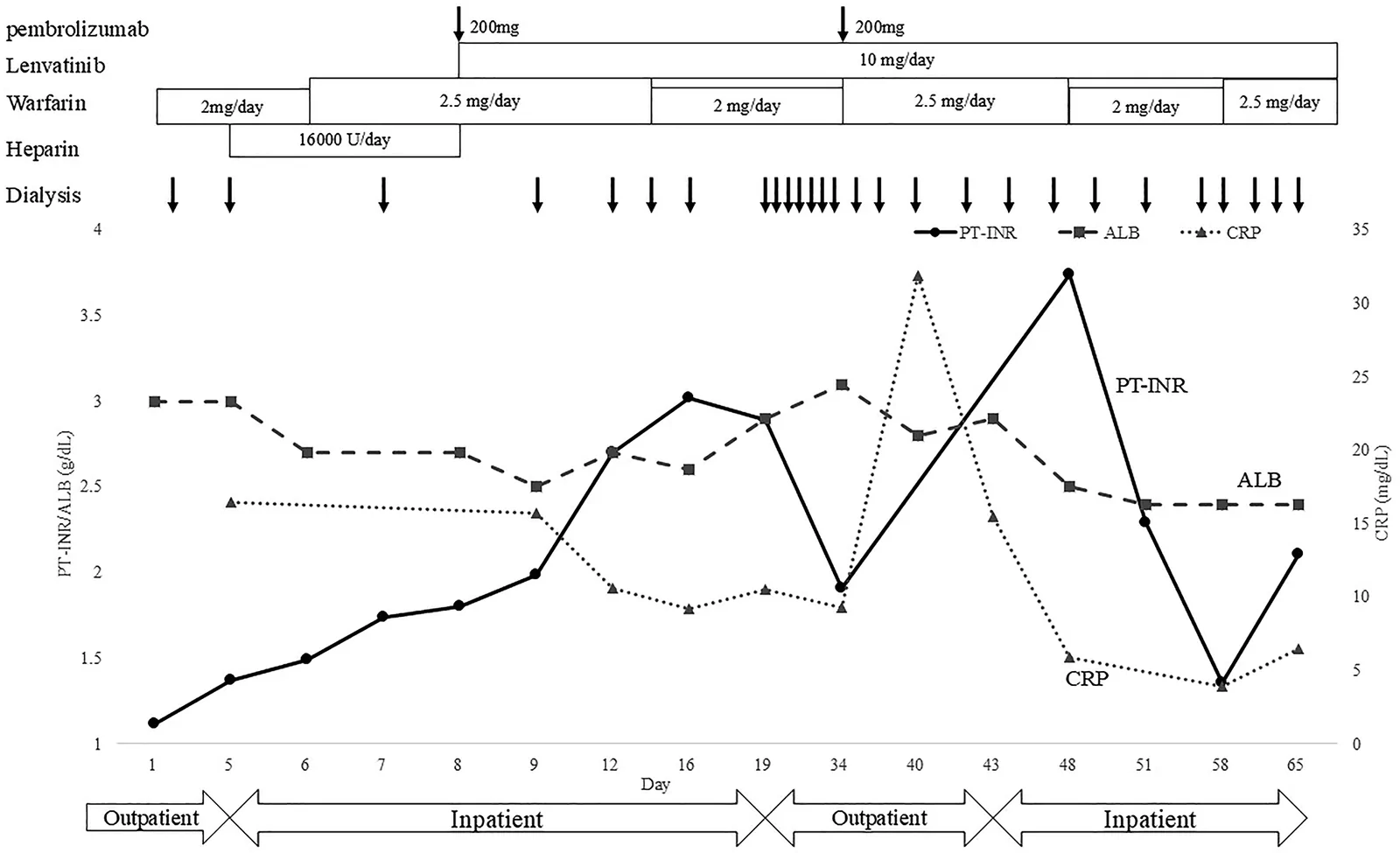
Treatment course with pembrolizumab, lenvatinib, warfarin, and heparin, and changes in PT-INR, albumin, and CRP levels. Day indicates the number of days from warfarin initiation. PT-INR and albumin are shown on the left axis, and CRP on the right axis. Combination therapy with lenvatinib and pembrolizumab was initiated on day 8 after warfarin initiation. The patient underwent dialysis three times weekly. PT-INR: prothrombin time-international normalized ratio, CRP: C-reactive protein

**Fig. 4:**
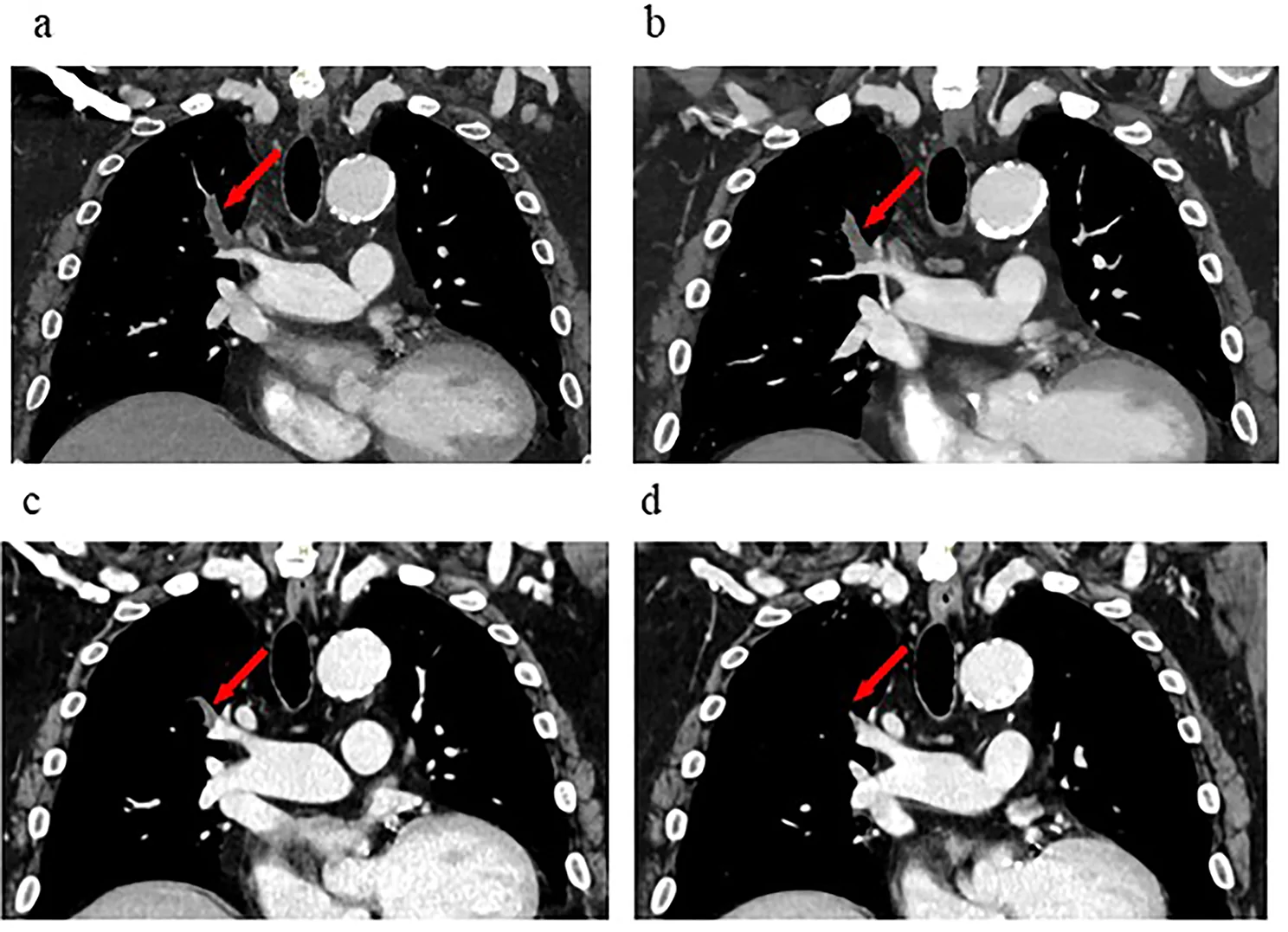
Abdominal CT after starting warfarin treatment. (**a**) Days 5, (**b**) 19, (**c**) 43, and (**d**) 133. Abdominal CT showed a reduction of right pulmonary artery embolism. CT: computed tomography

Warfarin was initiated at 2 mg/day for thrombosis prophylaxis (Fig. 3), with a target PT-INR range of 1.6–2.6. On day 5 after warfarin initiation, the patient was admitted to the hospital with complaints of exertional dyspnea and fatigue. Laboratory testing revealed a D-dimer level of 6.0 µg/mL (Table 1). Contrast-enhanced CT scan showed a right pulmonary artery embolism (Fig. 4a), and the patient was hospitalized. Heparin sodium injection was started at 16,000 U/day to treat right pulmonary artery embolism. At that time, the PT-INR was 1.37. Consequently, the warfarin dose was increased to 2.5 mg/day the following day. Dyspnea improved by day 8 of warfarin administration.

**Table 1:** Blood examination data on admission

	Values
BUN, mg/dL	28.2
CRE, mg/dL	5.77
TP, g/dL	7
ALB, g/dL	3
T-Bil, mg/dL	0.4
AST, U/L	17
ALT, U/L	16
WBC, /μL	9,800
Neut, /μL	8,820
Hb, g/dL	8.4
PLT, /μL	24.3 × 10^4^
CRP, mg/dL	16.5
LDH, U/L	217
KL-6, U/mL	275
D-Dimer, μg/mL	6
PT-INR	1.37
APTT, seconds	47.6

BUN: blood urea nitrogen, CRE: serum creatinine, TP: total protein, ALB: albumin, T-Bil: total bilirubin, AST: aspartate aminotransferase, ALT: alanine aminotransferase, WBC: white blood cell, Neut: neutrophil, Hb: hemoglobin, PLT: platelet, CRP: C-reactive protein, LDH: lactate dehydrogenase, APTT: activated partial thromboplastin time

Combination therapy with lenvatinib (10 mg/day) and pembrolizumab (200 mg every 3 weeks) was initiated on day 8 of warfarin administration. At that time, the PT-INR was 1.80, within the target therapeutic range, and heparin sodium was discontinued. On day 12 of warfarin (day 5 of lenvatinib), the PT-INR elevated to 2.70. By day 14, the warfarin dose was reduced to 2 mg/day due to concerns about excessive prolongation. The PT-INR subsequently measured 3.02 on day 16 (day 9 of lenvatinib) and 2.89 on day 19 (day 12 of lenvatinib). C-reactive protein (CRP) was markedly elevated at admission (16.5 mg/dL; Table 1) but gradually decreased, reaching 9.2 mg/dL by day 16 of warfarin administration (Fig. 3). CT imaging revealed a slight reduction in the size of the right pulmonary artery thrombus (Fig. 4b). No worsening of respiratory distress or adverse events attributable to lenvatinib were observed, and the patient was discharged. He was advised to avoid vitamin K–rich foods such as green vegetable juice, natto, and chlorella, and he reported adhering to these instructions. Albumin levels remained relatively stable (2.5–3.0 g/dL) throughout the first hospitalization (Fig. 3).

During an outpatient visit on day 34 of warfarin administration (day 27 of lenvatinib), the PT-INR was 1.91, prompting an increase in the warfarin dose to 2.5 mg/day. On day 40 of warfarin administration (day 33 of lenvatinib), the patient developed anorexia, dysgeusia, general fatigue, and abnormal gait and visited a local clinic. Laboratory tests revealed marked inflammation, with a white blood cell count of 14,360/µL and a CRP level of 31.9 mg/dL. Three days after the initial visit (on day 43 of warfarin administration) to the local clinic, the patient was admitted to our hospital for further evaluation. CT scan revealed a slight reduction in the size of the right pulmonary artery thrombus (Fig. 4c), with no apparent changes in the left RCC and no obvious infectious foci. During the outpatient period, albumin levels ranged from 2.9 to 3.1 g/dL.

At admission, the patient’s body temperature was 36.4 °C, white blood cell count was 10,400/µL, and CRP was 15.5 mg/dL. The elevated inflammatory markers were considered tumor-related. Thus, no antibiotics were administered during the patient’s hospital stay. At that time, the albumin level was 2.9 g/dL (Fig. 3). On day 48 of warfarin administration (day 41 of lenvatinib), the PT-INR increased to 3.74, prompting a reduction of the warfarin dose to 2 mg/day. Fatigue improved by day 50 of warfarin administration (hospitalization day 5), and the patient was able to fully consume a dialysis diet (1600 kcal, 6 g salt) with vitamin K-restricted meals (130–230 µg/day). During the second hospitalization, albumin levels ranged from 2.4 to 2.5 g/dL. Warfarin was subsequently adjusted to 2 and 2.5 mg/day, and the patient was discharged on day 65 of warfarin administration (day 58 of lenvatinib).

Warfarin treatment successfully controlled the pulmonary embolism (Fig. 4b–d), and the patient occasionally experienced exertional dyspnea but was managed as an outpatient. On day 80 of lenvatinib administration, disease progression was noted, and cabozantinib was initiated at 40 mg/day as second-line treatment. At the start of cabozantinib, the warfarin dose was 2 mg/day, with a PT-INR of 2.13. After 7 days, the PT-INR increased to 3.14, prompting a reduction of the warfarin dose to 1.5 mg/day. The patient was transferred to another hospital on day 10, and follow-up was discontinued. During cabozantinib treatment, albumin levels ranged from 1.7 to 1.8 g/dL. No bleeding events were observed during treatment.

### Discussion and conclusions

In this report, the patient developed renal vein and inferior vena cava tumor thrombus from a left RCC, indicating a high risk of thrombus formation [5]. The incidence of venous thromboembolism is high among patients on dialysis [22, 23].

Patients on hemodialysis who undergo treatment three times per week while receiving warfarin therapy are at increased risk of bleeding [24]. Strict PT-INR control is therefore essential. In the present case, PT-INR prolongation was observed after the initiation of combination therapy with lenvatinib and pembrolizumab during ongoing warfarin therapy under conditions of hypoalbuminemia.

The potential inhibition of CYP2C9 by lenvatinib should be considered. Sorafenib has been reported to increase PT-INR and bleeding risk when co-administered with warfarin, likely due to competitive inhibition of CYP2C9 [25]. According to FDA labeling, sorafenib inhibits CYP2C9 with a Ki value of approximately 7–8 µM, and its clinical maximum plasma concentration (Cmax) is in a similar range, resulting in an [I]/Ki ratio approaching unity and suggesting possible in vivo inhibition. In comparison, lenvatinib exhibits an IC₅₀ of > 100 µM for CYP2C9 inhibition, whereas its clinical Cmax at a 24-mg dose is approximately 1.6 µM. The resulting [I]/IC₅₀ ratio is well below 0.1, indicating a low likelihood of clinically relevant CYP2C9-mediated interactions.

The patient exhibited markedly elevated CRP levels, indicating systemic inflammation. CYP2C9 is responsible for the metabolism of warfarin [26], and inflammation can downregulate the expression and activity of CYP enzymes. Although a decline in CRP levels was observed over time during the initial hospitalization (Fig. 3), suggesting the recovery of CYP activity, PT-INR continued to increase.

These findings suggest that CYP2C9-related interactions, although theoretically limited under normal conditions, may have become clinically relevant in the presence of systemic inflammation, thus contributing to PT-INR prolongation.

PT-INR elevation associated with cabozantinib has been reported [27]. Cabozantinib as well as lenvatinib is a tyrosine kinase inhibitor; cabozantinib exhibits a high protein-binding rate (≥ 99%) [28] and shares similar pharmacokinetic characteristics with lenvatinib. Previous reports primarily discuss metabolic interactions, particularly involving CYP enzymes. Cabozantinib has also been suggested to possess metabolic interaction potential through CYP-related mechanisms.

In the present case, a rapid increase in PT-INR was also observed after cabozantinib initiation, suggesting that tyrosine kinase inhibitor-related effects may have contributed, although the exact mechanism remains unclear.

Hypoalbuminemia is common in patients undergoing long-term hemodialysis [29]. In the present case, albumin levels were low (2.5–3.0 g/dL) at the time of the first hospitalization. Albumin serves as the primary binding protein for many drugs, and reduced albumin levels can limit drug-binding capacity [30, 31], particularly when albumin levels fall below 2.5 g/dL.

Competitive binding between warfarin and lenvatinib may theoretically increase the unbound warfarin fraction. However, for low-extraction-ratio drugs such as warfarin, alterations in protein binding are typically compensated by corresponding changes in clearance, resulting in minimal effect on steady-state unbound drug concentrations. Therefore, only protein-binding displacement is unlikely to account for the observed PT-INR prolongation and is better interpreted as a contributory [32].

Daily vitamin K intake during hospitalization was controlled at 130–230 µg/day, which is considered to provide stable anticoagulation [33], suggesting a minimal impact of diet on PT-INR. The patient developed known adverse effects of lenvatinib therapy, including dysgeusia and anorexia, which likely led to reduced dietary intake, including vitamin K. Given that vitamin K plays a critical role in the synthesis of coagulation factors, decreased intake might have increased warfarin sensitivity.

Although the warfarin dose was adjusted to alternate between 2.0 and 2.5 mg/day in the present case, a more finely titrated regimen, such as an intermediate daily dose (e.g., 2.25 mg/day), might have provided more stable anticoagulation control.

Concomitant use of warfarin and combination therapy with lenvatinib and pembrolizumab in patients with RCC undergoing hemodialysis may enhance anticoagulant effects via multiple interacting factors, including CYP-related interactions, systemic inflammation, and hypoalbuminemia. Therefore, careful follow-up, including more frequent outpatient visits, is necessary for appropriate PT-INR management.

## Data Availability

The datasets generated during the current study are available from the corresponding author on reasonable request.

## Abbreviations

APKD: Acquired polycystic kidney disease
RCC: Renal cell carcinoma
PT-INR: Prothrombin time-international normalized ratio
DOACs: Direct oral anticoagulants
CYP: Cytochrome P-450
CT: Computed tomography
